# Structure‐Based Discovery of Coumarin‐Triazole Derivatives Targeting the Conserved QGDNQ Motif of Chandipura RdRp

**DOI:** 10.1155/sci5/1713642

**Published:** 2026-11-10

**Authors:** Anjana C. Lalu, Varun Thachan Kundil, Mukhtar Ahmed, Bristow Ben Joseph, Amritha Thaikkad, Radul R. Dev, Muthipeedika Nibin Joy, Rajesh Raju, Abhithaj Jayanandan, Grigory V. Zyryanov

**Affiliations:** ^1^ Centre for Integrative Omics Data Science (CIODS) Yenepoya (Deemed to be University) Mangalore Karnataka 575018 India; ^2^ Department of Zoology College of Science King Saud University P.O. Box 2455 Riyadh 11451 Kingdom of Saudi Arabia ksu.edu.sa; ^3^ Laboratory of Advanced Materials Green Methods and Biotechnology Institute of Chemical Technology Ural Federal University Ekaterinburg Russia urfu.ru; ^4^ I. Ya. Postovskiy Institute of Organic Synthesis Ural Division of the Russian Academy of Sciences 22 S. Kovalevskoy Street Yekaterinburg 620219 Russia

**Keywords:** Chandipura virus, coumarin-triazole derivatives, molecular dynamics, RNA-dependent RNA polymerase structural prediction, virtual screening

## Abstract

Chandipura virus (CHPV) is a recently discovered neurotropic virus associated with severe encephalitis in children and for which there is currently no specific antiviral treatment. The viral RNA‐dependent RNA polymerase (RdRp) is an attractive antiviral target because of its essential role in viral RNA synthesis and the presence of conserved catalytic motifs required for polymerase function. In this study, a structure‐based computational approach was employed to investigate coumarin‐triazole derivatives as potential inhibitors targeting the conserved QGDNQ motif‐containing region of CHPV RdRp. A homology model of CHPV RdRp was generated and structurally evaluated, followed by prediction of the ligand‐binding pocket. A set of 80 coumarin‐triazole derivatives was subjected to extra‐precision (XP) molecular docking, and compounds 7a, 7b, and 7d were identified as potential candidates based on their docking scores and predicted binding free energies. Compound 7a showed a favorable docking score and interactions with the residues close to the catalytic motif, especially ASN705. The ADMET analysis also indicated favorable physicochemical and pharmacokinetic properties for the selected compounds. Molecular dynamics simulations were subsequently performed for 200 ns for both the protein‐only system and the ligand‐bound complexes to assess their structural stability and conformational behavior. RMSD and RMSF analysis revealed that Compound 7a exhibited comparatively lower residue fluctuations among the ligand‐bound systems. PCA indicated a more compact conformational distribution comparable to that observed for the remdesivir‐bound system. Post‐MD MM/GBSA analysis further supported the favorable binding of Compound 7a. Overall, the integrated computational analysis identified Compound 7a as a promising coumarin‐triazole candidate for further investigation against CHPV RdRp. Experimental studies are required to validate its antiviral activity and mechanism, and the findings provide a basis for further optimization of the coumarin‐triazole scaffold toward potential non‐nucleoside CHPV RdRp inhibitors.

## 1. Introduction

Chandipura virus (CHPV) is a newly discovered zoonotic pathogen that poses a serious threat to public health, especially in the Indian subcontinent [1]. It belongs to the Vesiculovirus genus within the Rhabdoviridae family. CHPV is transmitted by the sand fly species *Phlebotomus* sp. and *Sergentomyia* sp. as well as by the mosquito *Aedes aegypti* [2]. It was first discovered in 1965 when Bhatt and Rodriguez, two researchers at the Virus Research Centre (VRC) in Pune, made an unexpected discovery. They were in the northern Maharashtra village of Chandipura, which is near Nagpur. The village where it was initially discovered gave the virus the name “Chandipura” [3].

During the 2024 outbreak, the World Health Organization (WHO) reported 245 cases of acute encephalitis syndrome (AES) in India between early June and August 15, 2024, including 82 deaths (33% case fatality rate); 64 of these cases were identified as CHPV infections, representing the largest reported CHPV outbreak in two decades. CHPV, which is endemic to India, typically causes AES outbreaks during the monsoon season and is transmitted through sandflies, mosquitoes, and ticks. Historically, CHPV infection has been associated with case fatality rates ranging from 56% to 75% [1].

It encodes five structural proteins, namely, nucleocapsid protein (N), phosphoprotein (P), matrix protein (M), glycoprotein (G), and RNA‐dependent RNA polymerase L (L). L protein codes for the biggest viral protein, which has 2092 amino acids and weighs 238.5 kDa [4]. The L protein, together with the P protein, forms the viral polymerase complex responsible for viral RNA transcription and replication and therefore represents an important target for antiviral development [5–7]. A highly conserved QGDNQ motif (residues 702–706) within the CHPV L protein is located in the catalytic region of the RdRp and represents a functionally important region for viral RNA synthesis [8, 9]. The functional importance and conservation of this region make the QGDNQ‐containing pocket a potentially valuable site for structure‐based antiviral discovery [8].

Studies on mononegaviruses have shown that the conserved QGDNQ motif forms the key catalytic center of RdRp and plays a vital role in viral RNA synthesis [10]. Research on viruses like vesicular stomatitis virus, rinderpest, and Nipah has shown that alterations in this motif can entirely hinder polymerase function, underscoring its critical importance [11]. However, there are currently no authorized vaccines or antiviral drugs for CHPV infection [12] highlighting the need for the identification of novel therapeutic candidates [12].

Natural products are a major source of chemical diversity and form an essential component of modern pharmaceuticals [13]. Coumarins are naturally occurring derivatives of benzopyrone that exhibit a variety of pharmacological effects and have garnered significant attention as essential structures for drug development [14]. Their structural versatility and broad biological activities have attracted significant attention in medicinal chemistry for the development of novel therapeutic agents. Coumarin derivatives exhibit a broad range of biological activities, including antibacterial, anti‐inflammatory, and antiviral properties, and have been investigated for various therapeutic applications, such as photochemotherapy, anticancer, and anti‐HIV therapies [15–18]. Several naturally occurring coumarin‐based antibiotics, including novobiocin, clorobiocin, and coumermycin A1, further highlight the therapeutic potential of this chemical scaffold [19]. Their antiviral activity has been reported against a range of viruses, including human immunodeficiency virus (HIV), influenza virus, hepatitis B and C viruses (HBV and HCV), dengue virus (DENV), and chikungunya virus (CHIKV) [20–24]. Mechanistic studies indicate that coumarin derivatives interact with various crucial viral proteins, including proteases, reverse transcriptases, integrases, and RNA‐dependent RNA polymerases highlighting the versatility of the coumarin scaffold in antiviral drug discovery [20, 25].

Despite these advancements, the potential of coumarin derivatives to target the conserved QGDNQ motif of CHPV RdRp has not been specifically investigated to date. This gap highlights the rationale for the present study. The high mortality associated with CHPV infections and the absence of authorized vaccines or antiviral therapies underscore the urgent need for novel therapeutic approaches. Natural products are valuable sources of antiviral agents, owing to their inherent biological compatibility and extensive chemical diversity, which enable effective interactions with viral targets while exhibiting lower toxicity. In this context, structure‐based drug discovery approaches offer a robust and effective foundation for identifying potential inhibitors for the CHPV.

## 2. Methodology

Due to the lack of an experimentally determined crystal structure for the CHPV RdRp protein, computational modeling methods were employed [26]. Molecular docking using Schrödinger Maestro was employed to evaluate the binding affinities and interactions between Coumarin derivatives and the target protein, followed by molecular dynamics (MD) simulations to analyze the stability and dynamic behavior of the resulting protein‐ligand complexes.

### 2.1. Homology Modeling

As no experimentally determined crystal structure is currently available for CHPV RdRp, homology modeling was employed to generate its three‐dimensional structure for subsequent screening studies. Homology modeling relies on the concept that proteins with similar amino acid sequences tend to have similar three‐dimensional structures, allowing a reliable structural model to be generated using a closely related template. The amino acid sequence of the CHPV RdRp (UniProt ID: P13179) was utilized to search for suitable template structures in the SWISS‐MODEL Template Library (SMTL) using BLAST. The identified templates were evaluated and sorted according to their Global Model Quality Estimation (GMQE) scores, with the top‐ranked template selected to develop the 3D model for further analysis.

Based on this analysis, the highest‐scoring template was selected for structural modeling, and the target sequence was subsequently aligned with the template to generate the three‐dimensional model. Following the selection of the best template, the target‐template alignment was refined to ensure the accurate placement of insertions and deletions. A conformational search was performed to model the loop regions and reconstruct structurally variable segments, while the conserved regions were directly adopted from the template structure [27]. The resulting model was subjected to structural quality assessment using multiple complementary validation parameters. The model was evaluated using the ERRAT analysis [28], ProSA analysis [29], and Ramachandran plot, Global Model Quality Estimate (GMQE), QMEANDisCo Global score, and QMEAN4 score provided by SWISS‐MODEL, to assess its overall structural quality and stereochemical properties. QMEAN‐based assessments provided estimates of the overall model quality, while ERRAT assessed the quality of nonbonded atomic interactions. ProSA was used to evaluate the overall energy profile of the modeled structure. The Ramachandran plot analysis evaluates the stereochemical integrity of the protein by assessing the backbone dihedral angles, phi (φ) and psi (ψ). Residues situated in favored regions suggest a reliable model, while those located in disallowed areas point to possible structural inconsistencies [30]. These complementary assessments were used to evaluate the suitability of the generated model for subsequent structural analyses.

### 2.2. Protein Preparation

The structure of the target protein, which was modeled using homology, underwent preprocessing through the Protein Preparation Wizard of the Schrodinger Suite (Version 14.1.138) [27, 31]. Throughout the preprocessing phases, disulfide bonds, bond orders, and formal charges were designated to ensure correct chemical states. The stability of the structure was enhanced by introducing hydrogen atoms and the protonation states of ionizable residues at a physiological pH of 7.4 ± 2.0 were assessed using the PROPKA module. The OPLS4 force field was selected for its superior ability to accurately model interactions between proteins and ligands, as well as small‐molecule solvation, which in turn boosts the dependability of docking and simulation outcomes [32]. The Impref utility was utilized to carry out restrained energy minimization, optimizing the positioning of heavy atoms and improving the overall structural geometry. “H‐bond assignment” tool was used to adjust the hydrogen‐bonding network. To minimize the energy of the structure, the OPLS4 force field is utilized.

### 2.3. Active Site Prediction and Grid Generation

Although the catalytic site was already known based on the conserved QGDNQ motif, additional binding site prediction using SiteMap and PrankWeb was performed for the validation of site in predicted structure and to assess its druggability, ensuring the reliability of the selected docking region. With default parameters in Schrödinger’s SiteMap, potential binding sites on the CHPV RdRp L protein have been explored and characterized. SiteMap examines the entire protein to find possible ligand binding sites and evaluates the druggability of these sites [33]. An additional tool for predicting binding sites based on independent structural analysis, PrankWeb (P2Rank) [34], was employed to provide further validation for the identified catalytic site. The conserved catalytic motif QGDNQ (GLN702, GLY703, ASP704, ASN705, and GLN706), forming the active site of the polymerase, was initially identified. To further confirm the conservation of the QGDNQ motif, multiple‐sequence alignment of the CHPV RdRp L protein and representative rhabdovirus L‐protein sequences was performed independently using Clustal Omega [35] and MAFFT [36]. A receptor grid was then generated around this area to define the docking area. The design centered around this motif and a receptor grid measuring 20 × 20 × 20 Å were created with Schrödinger’s receptor grid generation module, encompassing the active site residues of the CHPV RdRp L protein. In addition to defining the ligand‐binding region, the grid also incorporates the physicochemical properties of the receptor, including interaction potentials based on force field calculations. These Glide receptor grids facilitate the docking process by allowing a systemic exploration of ligand conformations within the binding site to determine the most advantageous receptor‐ligand complexes [37].

### 2.4. Proposed Synthesis of Coumarin‐Triazole Hybrids 7a–d and Ligand Preparation

Considering the known antiviral properties and structural adaptability of the coumarin framework, coumarin‐derived compounds offer a promising basis for creating new antiviral drugs. The synthesis of intermediates **3** and **4** can be carried out by a previously reported procedure [19, 38]. The synthesis of intermediates **6a–d** can be performed by copper‐catalyzed azide‐alkyne cycloaddition (CuAAC) reaction of coumarin intermediate **4** with different azides **5a–d** as reported by Joy et al. [39]. Azide **5a** (5‐azidobenzene‐1,2,3‐triol) can generate the intermediate **6a**, azide **5b** (4‐azidobenzene‐1,2‐diol) can form intermediate **6b**, azide **5c** (5‐(azidomethyl)benzene‐1,2,3‐triol) generates intermediate **6c,** and **6d** can be obtained from azide **5d** (4‐(azidomethyl)benzene‐1,2‐diol). The intermediates **6a-d** can be further subjected to hydrolysis by a previously reported protocol to obtain the final compounds **7a-d** [40]. The complete synthetic procedures and reaction conditions are provided in the Supporting File 1. For computational screening, a virtual library comprising 80 coumarin‐triazole derivatives was prepared based on the coumarin‐triazole scaffold and the structural variations considered in the study. The 2D structures of the ligand set were initially drawn using ChemBioDraw 14 software, and the generated .cdx files were subsequently imported into Maestro for further ligand preparation. Using the Schrodinger suite’s LigPrep module, all of the metabolites were preprocessed. Potential ionization and tautomeric states were produced using the Epik Classic module at a target pH of 7.0 ± 2.0, which represents physiological conditions [41]. All possible tautomers were generated and chiralities were determined from the 3D structures.

### 2.5. Molecular Docking and Binding Free‐Energy Calculation

Molecular docking was performed using the Glide module of the Schrödinger Suite to evaluate the binding interactions of the prepared coumarin‐triazole derivatives within the predicted binding pocket of the CHPV RdRp L protein [42]. A total of 80 prepared ligands were directly subjected to Glide Extra Precision (XP) docking [13]. The lead compounds were screened following docking, and the compounds with the best docking scores were selected for further study. According to the XP docking data, the binding free energy was also calculated using the Prime module of the Schrödinger suite’s MM‐GBSA (molecular mechanics generalized born surface area). The top compounds were selected based on their docking scores and binding free energy. Remdesivir has been reported to have antiviral activity against CHPV and has previously been evaluated as a reference compound in computational studies of CHPV [9, 43]. Remdesivir, an ATP analog and a potent inhibitor of Ebola virus, currently used in emergency clinical settings for COVID‐19, is hypothesized to also bind CHPV RNA‐dependent RNA polymerase (RdRp) and inhibit viral replication. It was used as a reference inhibitor to assess and compare the in silico binding affinity and efficiency of candidate compounds [9].

### 2.6. ADMET Analysis

In silico ADMET (absorption, distribution, metabolism, excretion, and toxicity) prediction was used to assess the pharmacokinetic and toxicity properties of the selected drugs. This method enables the early assessment of drug safety and likeness while also reducing all of the costs and time related to experimental testing. Drug likeness was evaluated using physical properties such as molecular weight, hydrogen‐bond donors and acceptors, lipophilicity, and oral bioavailability, as well as known criteria such as Lipinski’s rule of five. Lack of ADMET features is a major reason why many clinical studies end in failure [44]. Using the AI Drug Lab platform, SMILES representations of the selected compounds were retrieved from the PubChem database to evaluate their pharmacokinetic and pharmacodynamic properties [45–47]. All of the compounds across multiple ADMET categories were systematically evaluated using these computational analyses. First, physicochemical and drug‐likeness criteria were used to screen out unsuitable compounds. Key pharmacokinetic characteristics such as oral bioavailability, permeability across the blood‐brain barrier, and binding to plasma membranes were analyzed to assess their ability for absorption and distribution. The toxicity‐related factors evaluated included Ames mutagenicity, acute toxicity, and liver damage caused by drugs, which were used to exclude compounds exhibiting unfavorable safety profiles and to prioritize candidates with lower predicted toxicity risks.

### 2.7. Molecular Dynamic Simulation

MD simulations were performed using the Desmond module implemented in the Schrödinger software suite [41]. The stability and adaptability of protein‐ligand complexes were analyzed through MD simulations. MD assesses the physical movements of atoms and molecules within the docked complex of the protein and ligand to ensure structural stability and identify conformational alterations [48]. The chosen protein‐ligand complexes underwent MD simulations to assess their stability. The protein‐ligand complex was situated within a TIP3P water box, and water molecules were substituted with Na^+^ and Cl‐ ions to neutralize the reaction. For short‐range van der Waals and electrostatic interactions, a cutoff of 9.0 Å was employed, and periodic boundary conditions were imposed in all directions. The system was equilibrated under both NVT and NPT ensembles using a Berendsen thermostat and barostat, following a multistep relaxation process that included both restrained and unrestrained energy minimization, with simulations carried out at 10 K for 12 ps and 300 K for 24 ps. The MD simulation was carried out for 200 ns under the NPT ensemble at 300 K and 1.013 bar pressure, using a Nose‐Hoover thermostat and a Martyna‐Tobias‐Klein barostat, with a time step of 2 fs [49]. During the simulations, 2000 frames were captured for every system, and the OPLS4 force field was employed to minimize energy. Following the 200 ns MD simulations, the final 500 frames from each trajectory were extracted and used for MM/GBSA binding free‐energy calculations using the thermal_mmgbsa.py script. The stability of the complex was examined using the ligand interactions, the root mean square deviation (RMSD), and the root mean square fluctuation (RMSF), and principal component analysis (PCA) was performed.

## 3. Result

### 3.1. Homology Modelling

Homology modeling of the CHPV RdRp was performed using the SWISS‐MODEL due to the absence of an experimentally resolved crystal structure. Using the amino acid sequence of RdRp (UniProt ID: P13179) as input, suitable template structures were identified by aligning them with Protein Data Bank (PDB) entries. Previous studies have reported that sequence identities of ≥ 40% provide a favorable basis for comparative protein structure modeling [50]. The structure with PDB ID 6U1X, which is corresponding to the Vesicular Stomatitis Virus L protein, was selected from the available templates based on its sequence identity of 59.79% which exceeded the > 40% sequence‐identity benchmark for comparative modeling and with a favorable GMQE score of 0.81, supporting its suitability as a template. These parameters, in addition to alignment coverage (100%) and structural conservation, assisted in determining that 6U1X as the best template to generate the 3D model (Figure 1A) [51].

**FIGURE 1:**
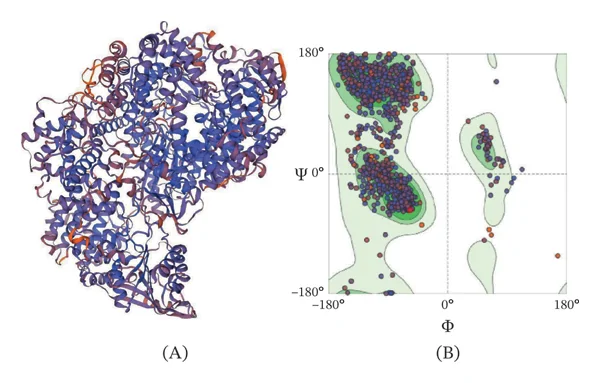
Homology‐modeled structure of the Chandipura virus RdRp protein. (A) Three‐dimensional ribbon representation of the homology model. Blue color represents regions with highest confidence and orange color denotes areas with lower confidence. (B) Ramachandran plot of the modeled structure.

According to the Ramachandran plot from SWISS‐MODEL (Figure 1B) indicated that the majority of residues occupied favored regions associated with stable secondary structures, while only a few residues were found in additionally allowed and disallowed regions. The observed distribution reflects favorable stereochemical characteristics, further supporting the reliability of the modeled protein backbone. The analysis showed that 95.04% of the residues were located in the most favorable regions, while 4.51% were found in additionally allowed regions and only 0.43% occurred in disallowed regions. The model exhibits strong reliability, with over 90% of residues located in favored regions.

Further structural validation was conducted using complementary parameters to assess model quality. The constructed model exhibited a GMQE score of 0.69 and a QMEANDisCo Global score of 0.75 ± 0.05, indicating a good overall predicted model quality. Additionally, the QMEAN4 score of −2.47 and ERRAT score of 92.55 further validated the model’s structural quality. The ProSA Z‐score of −19.06 suggested that the overall energy profile falls within the expected range for protein structures of comparable size. The stereochemical quality of the modeled structure was subsequently evaluated using Ramachandran plot analysis from SWISS‐MODEL. In order to investigate the modeling of the functionally important QGDNQ region more thoroughly, the target and the chosen template were compared at both the sequence and structural levels. The alignment revealed that the QGDNQ‐containing region was located correspondingly between the target and the template. Figure 2 illustrates the sequence and structural alignment of this region, offering further validation for the modeling of the QGDNQ‐containing segment utilized in later structural analyses.

**FIGURE 2:**
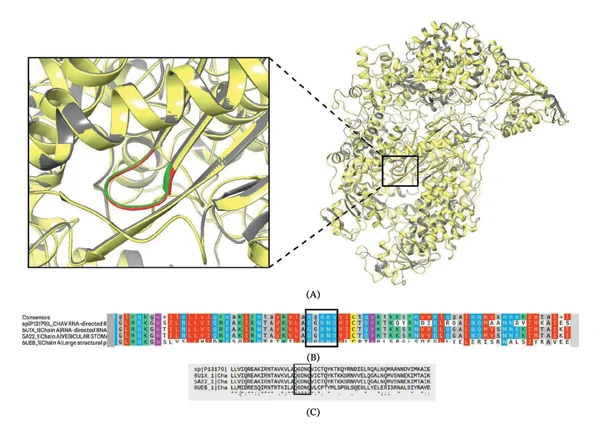
Structural and sequence alignment supporting the modeled QGDNQ motif region of CHPV RdRp. (A) Structural alignment of the modeled CHPV RdRp with the template structure, showing the corresponding spatial positioning of the QGDNQ motif region. Multiple‐sequence alignment showing conservation of the QGDNQ motif. Alignments generated using (B) MAFFT and (C) Clustal Omega.

In order to evaluate structural stability, MD simulation was conducted. An RMSD value under 3 Å is generally considered indicative of a reliable model [52]. During the 200 ns simulation, the modeled protein exhibited an average Cα RMSD of 2.785 ± 0.203 Å. The comparatively low standard deviation suggested that there was only a limited variation around the mean RMSD value during the simulation. The RMSD trajectory displayed an initial adjustment followed by a relatively stable pattern in the later phases of the simulation, suggesting that the overall protein fold was maintained throughout the simulation [44]. The findings, along with the evaluations of structural validation, supported the suitability of the modeled CHPV RdRp structure for subsequent active‐site identification and molecular docking studies.

### 3.2. Proposed Synthetic Route

The synthesis of compounds **7a–d** has been proposed after a detailed literature survey [19, 39]. As depicted in Figure 3, the classical Pechmann cyclization/condensation reaction of resorcinol **1** with β‐keto ester **2** in the presence of concentrated sulphuric acid can generate the coumarin intermediate **3**. This intermediate **3** can be treated with propargyl bromide in the presence of base (K_2_CO_3_) to obtain the *O*‐propargylated coumarin intermediate **4**.

**FIGURE 3:**
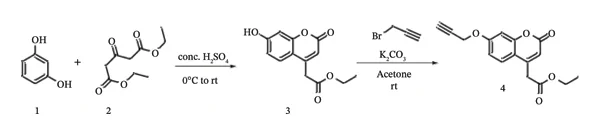
Synthetic route for obtaining intermediate **4**.

As detailed in Figure 4, the intermediate **4** can be subjected to copper‐catalyzed azide‐alkyne cycloaddition reaction (CuAAC) with different azides **5a–d** to get the coumarin‐triazole hybrid molecules **6a–d**. Finally, the ester group in intermediates **6a–d** can be hydrolyzed in the presence of a base like NaOH to generate the proposed coumarin‐triazole hybrids **7a–d**.

**FIGURE 4:**
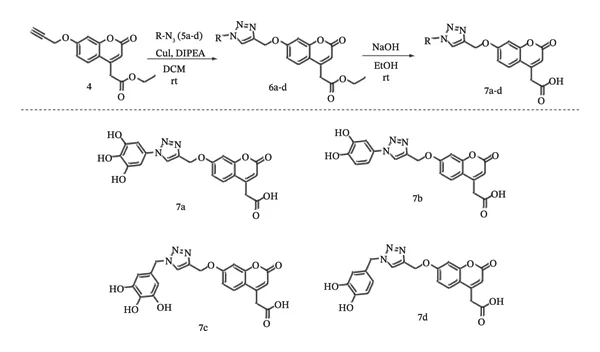
Synthetic route for obtaining final compounds **7a–d**.

### 3.3. Molecular Docking and Binding Free‐Energy Analysis

To verify the active site identified from the conserved QGDNQ motif and ensure the chosen docking region was reliable, SiteMap was used to detect potential ligand‐binding pockets and evaluate their suitability for drug binding. The binding site’s size, solvent exposure, tightness of interaction between site points and the receptor, balance between hydrophobicity and hydrophilicity, and degree of hydrogen bond between donation and acceptance are all described by the SiteScore that SiteMap generates [51].

A potential binding site is indicated by a SiteScore of more than one. All possible binding sites were found in the modeled protein, according to SiteMap analysis. Several possible binding pockets within the modeled protein were found by the SiteMap analysis, and some of them showed comparable SiteScore values. Among these, Binding site_2 (SiteScore = 1.046; Dscore = 1.003) was chosen because it lies close to the conserved QGDNQ motif, indicating that it is likely associated with the catalytic region and therefore relevant for ligand binding (Table 1). The selected binding pocket is illustrated in Figure 5.

**TABLE 1:** Scoring of binding sites from Sitemap.

Sl. No.	Site number	SiteScore	DScore
1	Binding_site_1	1.012	1.025
2	Binding_site_2	1.046	1.003
3	Binding_site_3	1.050	1.039
4	Binding_site_4	1.043	1.038
5	Binding_site_5	1.041	0.969

**FIGURE 5:**
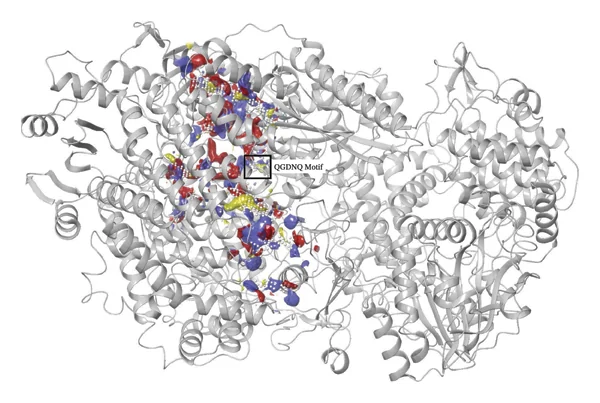
Predicted druggable sites in the RdRp of Chandipura virus identified using the SiteMap module.

Independent assessment using PrankWeb (P2Rank algorithm) identified the same region as the primary active site with the top‐ranked pocket exhibiting a high score (86.89), strong prediction probability (0.994), and a large residue composition, indicating a distinctly defined and biologically significant cavity. Significantly, this pocket contained residues next to the QGDNQ motif, highlighting its important role in the function of RNA polymerase. This motif found in the palm subdomain of the RdRp comprises residues GLN702, GLY703, ASP704, ASN705, and GLN706.

To further justify the selection of the docking site, the predictions from SiteMap and P2Rank were analyzed at the residue level. A combined total of 54 residues were identified in both predictions, accounting for 75% of the residues identified by SiteMap. Notably, both methods recognized the area around the conserved QGDNQ motif, specifically the residues from 702 to 706. SiteMap recognized residues 702 and 704–706, while P2Rank recognized residues 702–706 and residue 707. The complete lists of residues identified by SiteMap and P2Rank are provided in the Supporting Table 1-A and Supporting Table 1-B.

The predicted regions were further examined using surface representations of the modeled RdRp, demonstrating that the pockets independently identified by SiteMap and P2Rank occupied a common spatial region surrounding the QGDNQ motif (Figure 6). Structural alignment of the modeled CHPV RdRp with the template structure showed a corresponding spatial position of the QGDNQ motif region. In addition, target‐template sequence alignment around the QGDNQ region supported the correspondence of this conserved motif between the modeled CHPV RdRp and the template. This conserved sequence represents a characteristic signature of rhabdoviral polymerases and was clearly identified in the modeled structure.

**FIGURE 6:**
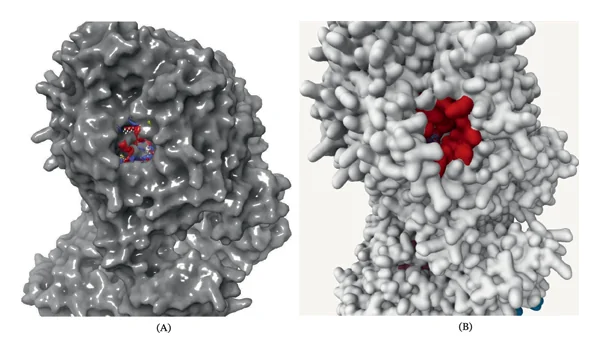
Predicted binding region of CHPV RdRp. Surface representations of the modeled RdRp showing the predicted binding pockets identified independently by (A) SiteMap and (B) P2Rank.

The biological relevance of this region is further supported by previous comparative studies of rhabdovirus L proteins by Cherian et al. and Mandal et al. which indicated that Block III, containing the QGDNQ sequence, is highly conserved and suggested that this pentapeptide region functions as an active site involved in either template recognition or phosphodiester bond formation. The QGDNQ sequence has also been reported as a conserved signature of rhabdovirus polymerases [8, 9]. Based on the convergence of independent pocket predictions, motif conservation, structural localization, and previously reported functional relevance, the QGDNQ‐containing region was selected as the docking site for subsequent analyses.

A total of 80 compounds were subjected to molecular docking. Compounds with favorable docking scores and binding free energies were analyzed further for their ligand‐protein interaction profiles, focusing specifically on hydrogen bonds and other beneficial interactions with residues located in the QGDNQ‐containing binding region. The compounds were initially evaluated based on their docking scores and favorable binding free energy values with the reference inhibitor serving as a comparative benchmark (docking score: −6.112 kcal/mol; MM‐GBSA binding free energy: −22.33 kcal/mol). Based on this comparative assessment, compounds 7a, 7d, and 7b were prioritized based on their relatively favorable predicted docking scores (−8.729, −8.496, and −8.028 kcal/mol, respectively), together with favorable MM‐GBSA binding free energies (−40.95, −41.36, and −38.39 kcal/mol, respectively) than the reference inhibitor.

Furthermore, all three compounds exhibited interactions with Asn705, a residue located within the conserved QGDNQ motif, along with other residues surrounding the predicted binding region. Based on this sequential evaluation of predicted docking scores, favorable binding free energies, and ligand‐protein interactions, compounds 7a, 7b, and 7d were prioritized for subsequent ADMET analysis and MD simulations. The docking scores and SMILES representations of all 80 compounds are provided in Supporting Table 1-C, while the detailed interactions of the selected compounds with CHPV RdRp are presented in Table 2 and Figure 7.

**TABLE 2:** Docking score and binding free energy of the selected drug leads.

Compound	Docking score	Binding free energy	Interacting residues
Remdesivir	−6.112	−22.33	TYR342, LYS525, ILE594, GLN706, LYS768
Base fragment	−5.756	−35.48	ASN284, TYR342, TYR596
Intermediate 3	−4.417	−35.89	ASN284, TYR342, ASN705, GLY767
Intermediate 4	−4.939	−35.41	ASN284, TYR342, LYS768
Intermediate 6a	−5.238	−52.14	ARG529, TYR596, TRP599, ASN600, GLU671, ASN705, GLY767
Compound 7a	−8.729	−40.95	ASN284, TYR342, ARG529, TYR596, TRP599, ASN600, GLU671, ASN705, GLY767, LYS768
Compound 7d	−8.496	−41.36	ASN284, TYR342, ASN600, GLU671, ASN705, GLY767, LYS768
Compound 7b	−8.028	−38.39	ASN284, TYR342, ASN600, GLU671, ASN705, GLY767

**FIGURE 7:**
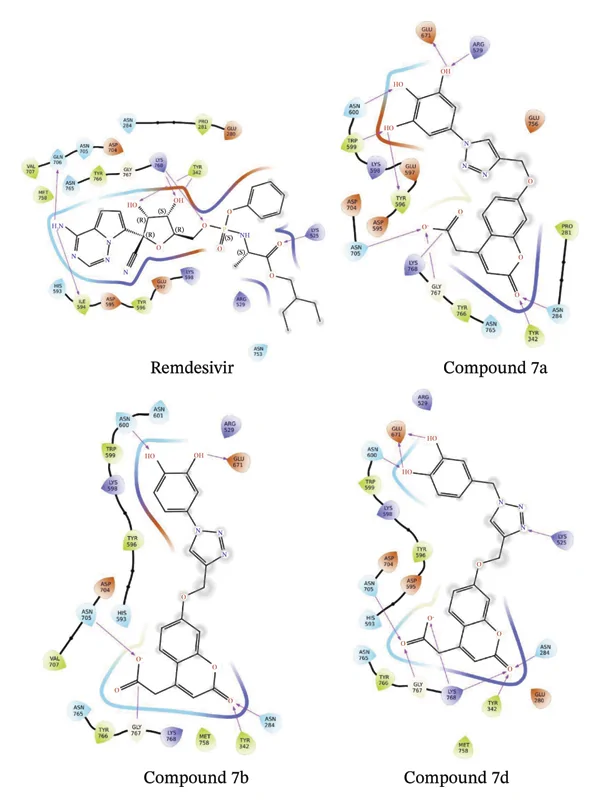
Ligand interaction diagram of RdRp of Chandipura virus with remdesivir and compound 7a, compound 7b, and compound 7d.

### 3.4. ADMET

The ADMET analysis provided preliminary predictions of the physicochemical, pharmacokinetic, metabolic, and toxicity‐related properties of the three selected compounds. The compounds showed molecular weights ranging from 408.08 to 424.08 g/mol, whereas remdesivir had a higher molecular weight of 602.23 g/mol. While all compounds contained 4 rings and 6–7 rotatable bonds, compared with remdesivir, which had 13 rotatable bonds and 13 hydrogen‐bond acceptors (Table 3). The predicted log KOW values vary from 0.36 to 0.72 and log D7.4 values falling between 1.86 and 1.94, suggesting a well‐balanced physicochemical profile. Caco‐2 permeability values were estimated (−5.53 to −5.48). According to the reference ranges provided in Table 3, the predicted human intestinal absorption (72.97%–73.59%) indicated values fall within the medium range. The predicted oral bioavailability values ranged from 43.87% to 44.41% which was comparable to the predicted value for remdesivir (44.0%), obtained using the same computational platform. However, there is no standardized threshold for oral bioavailability that determines the therapeutic effectiveness of antiviral drugs, and remdesivir is administered clinically by the intravenous route. Therefore, these values should be considered preliminary indicators of potential systemic exposure. These predictions provide a preliminary insight into the possible physicochemical properties and absorption characteristics of the compounds.

**TABLE 3:** ADMET Profiling of high‐scoring compounds.

Properties	Unit	Threshold	Compound 7a	Compound 7d	Compound 7b	Remdesivir
Molecular weight	g/mol	100∼600	424.08	422.1	408.08	602.23
Number of heteroatoms	/	1∼15	11	10	10	15
Number of rotatable bonds	/	≤ 11	6	7	6	13
Number of rings	/	≤ 6	4	4	4	4
Number of HA	/	≤ 12	11	10	10	13
Number of HD	/	≤ 7	3	2	2	4
log KOW	log‐ratio	0∼3	0.36	0.72	0.66	2.31
Caco‐2 permeability	Log (cm/s)	> −5.15	−5.53	−5.45	−5.48	−5.3
HIA	%	Poor: ≤ 30%; medium: 30%∼80%; optimal:≥ 80%	72.99	72.97	73.59	66.73
Pgp inhibition	%	Optimal: ≤ 30%; medium: 30%∼70%; poor: ≥ 70%	45.47	48.15	47.81	39.81
log D7.4	log‐ratio	1∼3	1.86	1.93	1.94	1.88
Aqueous solubility	log (mol/L)	Soluble: −2∼0; slightly soluble: −4∼−2; insoluble: < −4	−4.6	−4.58	−4.77	−3.58
Oral bioavailability	%		43.87	44.27	44.41	44
BBB	%	Optimal: ≤ 30%; medium: 30%∼70%; poor: ≥ 70%	13.93	10.86	12.73	22.42
PPBR	%	Optimal: ≤ 90%; poor: > 90%	59.13	59.2	58.57	45.19
VDss	L/kg	Optimal: 0.04∼20%; poor: otherwise	5.06	5.13	4.97	3.64
CYP2C9 inhibition	%		69.97	67.02	65.63	58.59
CYP2D6 inhibition	%		99.4	110.68	98.11	95
CYP3A4 inhibition	%		32.97	34.5	32.31	36.9
CYP2C9 substrate	%		32.66	32.96	32.44	34.69
CYP2D6 substrate	%		50.17	49.91	55.03	49.05
CYP3A4 substrate	%		34.84	38.0	36.26	38.8
Half life	hr		81.95	84.99	85.37	115.95
CL‐Hepa	uL min^−1^ (106 cells)^−1^		40.8	50.61	41.45	42.74
CL‐Micro	mL min^−1^ g^−1^		48.0	45.13	45.68	48.56
hERG blockers	%	Optimal: ≤ 30%; medium: 30%∼70%; poor: ≥ 70%	42.1	44.99	43.91	46.48
Ames	%	Optimal: ≤ 30%; medium: 30%∼70%; poor: ≥ 70%	46.09	46.6	45.84	42.68
DILI	%	Optimal: ≤ 30%; medium: 30%∼70%; poor: ≥ 70%	49.03	49.3	51.22	57.99
LD50	−log (mol/kg)		2.28	2.33	2.28	2.2

The distribution parameters indicated a moderate penetration across the blood‐brain barrier (10.86%–12.73%), plasma protein binding levels (58.57%–59.20%), and volume of distribution at steady state (VDss) values (4.97–5.13 L/kg), suggesting a similar distribution in tissues. The predicted BBB values fall within the optimal range, while the PPBR and VDss values also fall within their respective specified ranges. The compounds demonstrated relatively long predicted half‐lives ranging from 81.95 to 85.37 h, along with moderate hepatic clearance values between 40.80 and 50.61 μL min^−1^ (106 cells)^−1^ and microsomal clearance values from 45.13 to 48.00 mL·min^−1^·g^−1^. These values represent computational predictions of distribution and clearance‐related properties.

The toxicity‐related predictions showed relatively similar numerical values among the selected compounds. The predicted hERG blockade probabilities range from 42.10% to 44.99%, Ames mutagenicity scores are between 45.84% and 46.60%, DILI values range from 49.03% to 51.22%, and LD50 values range of 2.15–2.33. These computational toxicity predictions provide preliminary indicators for toxicity‐related profiling. The inhibition values for CYP2C9 varied between 65.63% and 69.97%, while those for CYP2D6 ranged from 98.11% to 110.68%, and the inhibition values for CYP3A4 fell between 32.31% and 34.50%. The projected substrate‐related values for CYP2C9, CYP2D6, and CYP3A4 were found to be within the ranges of 32.44%–32.96%, 49.91%–55.03%, and 34.84%–38.78%, respectively. These values represent model‐derived outputs generated by the prediction platform. In particular, values exceeding 100% should not be interpreted as inhibition greater than 100%. The CYP bioactivity data are based on a control‐normalized percentage activity scale, where 100% represents the standard control response, and values exceeding 100% signify responses that exceed the normalized control level [53].

In general, the ADMET evaluation provided an initial computational overview of the selected compounds concerning physicochemical, absorption, distribution, metabolic, and toxicity‐related characteristics. The predicted profiles showed compound‐dependent variations across the evaluated endpoints, with several parameters falling within the reference ranges. These findings support the use of ADMET prediction as a preliminary computational assessment during compound prioritization.

### 3.5. MD Simulation

#### 3.5.1. Root Mean Square Deviation (RMSD)

MD simulations were performed to evaluate the stability and dynamic behavior of the CHPV RdRp protein in both protein only and ligand‐bound states. Based on the docking scores, binding free energies, and ADMET (absorption, distribution, metabolism, excretion, and toxicity) characteristics of selected compounds, including compound 7a, compound 7b, and compound 7d, MD simulations were performed. We performed an RMSD analysis of the protein‐ligand complexes to assess conformational stability upon ligand binding. The protein RMSD analysis conducted during the 200 ns MD simulation revealed that the apo protein reached equilibrium within the first 10–20 nanoseconds and maintained a stable conformation for the remainder of the simulation, with only slight fluctuations. This indicates that the simulated RdRp structure remains fundamentally stable in the absence of ligand binding, providing a reliable benchmark for comparison with ligand‐bound systems. All protein‐ligand complexes also reached equilibrium within the initial 10–20 ns of the simulation and remained stable throughout the 200 ns trajectory, with no major conformational changes observed. The average protein RMSD values were 2.9646 Å for compound 7a, 3.095 Å for compound 7b, 3.0225 Å for compound 7d, and 2.760 Å for the reference ligand, remdesivir. The reference ligand remdesivir demonstrates the lowest RMSD values indicating stable interaction with the protein. Compared to compounds 7b and 7d, compound 7a displayed the lowest average RMSD, although the small differences among the compounds indicated broadly comparable conformational behavior. The slightly elevated RMSD values seen for compounds 7b and 7d may reflect minor differences in conformational fluctuations rather than a substantial difference in structural stability. The RMSD analysis gave a preliminary evaluation of the conformational dynamics of both the protein‐only and ligand‐bound systems over the course of the 200 ns simulations. The three complexes with ligands exhibited generally similar RMSD profiles, showing only minor variations in their average values. Figure 8 represents the average Cα RMSD plots of the RdRp‐ligand complex.

**FIGURE 8:**
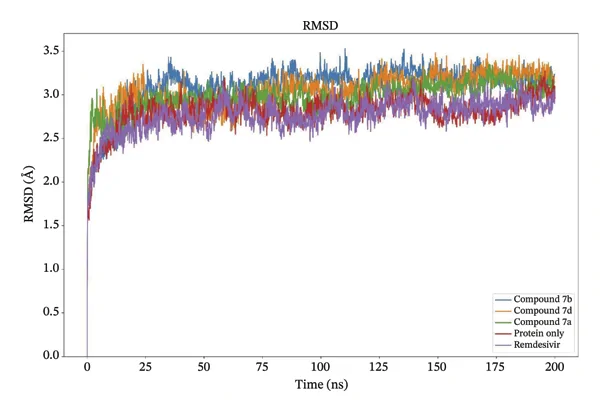
Cα RMSD profiles of CHPV RdRp in complex with remdesivir and compounds 7b, 7d, and 7a, along with the apo protein.

These findings offer an initial insight into the conformational dynamics of the complexes during the simulation and suggest that the ligand‐bound systems displayed comparable dynamic behaviors.

The ligand RMSD analysis showed relatively higher fluctuations during the initial 20 ns of the simulations for all compounds, suggesting an initial adjustment of the ligand conformations within the binding environment. Following this initial phase, the ligand RMSD profiles became comparatively stable, indicating that the ligands maintained relatively consistent conformations during the subsequent period of the simulations (Figure 9).

**FIGURE 9:**
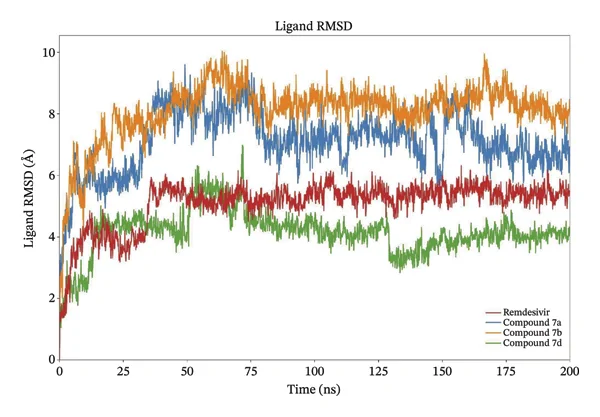
Ligand RMSD profiles of remdesivir and compounds 7b, 7d, and 7a.

#### 3.5.2. Root Mean Square Fluctuation (RMSF)

The root mean square fluctuation (RMSF) measures the average deviation of each atom from its mean position during a MD simulation. Higher RMSF values indicate greater mobility, while lower values suggest decreased mobility. The RMSF values for Cα across the 200 ns simulation period are displayed in Figure 10. Overall, RMSF analysis provides insight into the flexibility and stability of different regions of the protein. Lower fluctuations generally indicate more stable regions, whereas higher fluctuations reflect greater flexibility and dynamic movement. The RMSF profile of the protein‐only (apo) CHPV RdRp was first analyzed to assess the protein’s intrinsic flexibility in the absence of ligand binding. The apo protein exhibited relatively low fluctuations across most residues, with higher fluctuations primarily observed in terminal and loop regions, while the catalytic core remained stable during the entire simulation. The apo RMSF profile was subsequently used as a baseline for comparison with the residue‐level fluctuations observed in the ligand‐bound systems. The average mean Cα RMSF values were 1.174 Å for compound 7a, 1.2202 Å for compound 7b, 1.1893 Å for compound 7d, and 1.0881 Å for the reference ligand, remdesivir (Figure 10). Among the compounds examined, compound 7a exhibited the lowest average RMSF, followed by compound 7d and compound 7b. Among the compounds examined, compound 7a exhibited a slightly lower average RMSF than compound 7d and compound 7b. Although distinct fluctuation peaks were observed in the loop and terminal regions, they did not appear to affect the protein’s overall structural integrity. Overall, the RMSF profiles of the three ligand‐bound systems were broadly comparable, with only minor differences in their average residue fluctuations. These results offer an initial evaluation of the dynamic behavior at the residue level for the chosen complexes throughout the simulation, with compound 7a displaying a slightly lower average RMSF during the observed simulation timeframe.

**FIGURE 10:**
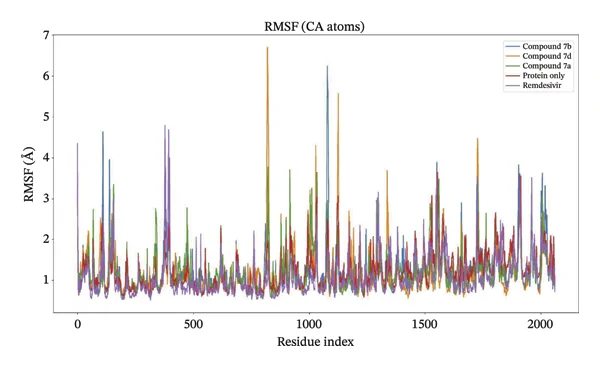
RMSF profiles of the Cα atoms of CHPV RdRp in the apo state and in complexes with remdesivir and compounds 7a, 7b, and 7d.

#### 3.5.3. Protein‐Ligand Interaction

Analysis of the interactions between CHPV RdRp and the ligands revealed several stabilizing contacts, including hydrogen bonds, hydrophobic interactions, and electrostatic forces (Figure 11). The interaction analysis showed that all three compounds maintained stable binding within the catalytic pocket over the course of the 200 ns MD simulation. Notably, all three compounds maintained interactions with the conserved catalytic QGDNQ motif (residues 702–706), which constitutes a key region of the active site of the viral RNA‐dependent RNA polymerase. Compound 7a exhibited the most advantageous interaction profile, maintaining stable interactions with residues in and around the catalytic motif, which ensured its orientation within the binding pocket remained consistent during the simulation. Compound 7a exhibited a highly persistent hydrogen‐bond interaction with Glu671 (99.0%), which was maintained throughout most of the 200 ns simulation. An interaction with Arg529 was also observed, with substantial occupancy (90.7%), although the interaction was more prominent during the initial ∼75 ns and became less frequent during the subsequent part of the trajectory. A water‐mediated interaction with Asp704 showed 41.85% occupancy and was observed intermittently throughout the simulation. Ionic interactions involving Asp595 (26.45%) and Gln756 (12.90%) were also detected. The persistence of all these contacts indicates that 7a remained associated with the RdRp binding region throughout the simulation. Compound 7b also maintained highly persistent hydrogen bonds with residues Glu671 and Lys768 (93.1% and 91.5%), accompanied by a prominent water‐mediated interaction. Compound 7d similarly retained a persistent interaction with Lys768 (90.95%), along with hydrogen‐bond interactions with Glu671, Met758, and Asn705. It also formed multiple water‐mediated interactions involving Asn704, Gln706, Asn705, and Asn765, indicating a broader network of direct and solvent‐mediated contacts. Among these, the interaction with Asn706 was particularly prominent during the latter part of the trajectory, while Asp704 and Asp705 showed recurrent contacts across the simulation period. Overall, the interaction profiles indicate that all three compounds remained stably associated with the catalytic pocket of CHPV RdRp throughout the 200 ns simulation. The persistence of hydrogen‐bond, ionic, and water‐mediated interactions, particularly with residues within or surrounding the conserved QGDNQ catalytic motif, supports the sustained positioning of the compounds within the active‐site region.

**FIGURE 11:**
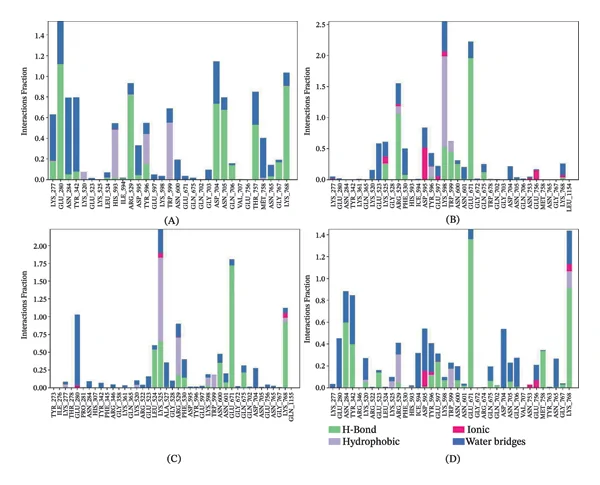
Protein‐ligand contact between (A) remdesivir and (B) compound 7b, (C) compound 7d, and (D) compound 7a.

#### 3.5.4. The Binding Free Energy of Post‐Molecular Dynamics

The binding free energy of particular poses from the MD trajectory was assessed using the MM‐GBSA method after the MD run. The final 500 frames of the 200 ns MD trajectory were selected for subsequent MM/GBSA analysis. Prior to conducting MD simulations, compound 7d demonstrated the most favorable binding free energy among the compounds screened (−41.36 kcal/mol), with compound 7a (−40.95 kcal/mol) and compound 7b (−38.39 kcal/mol) closely following. In contrast, remdesivir exhibited a notably weaker binding free energy (−22.33 kcal/mol). The binding free energy of compound 7a increased from −40.95 to –‐53.73 ± 4.08 kcal/mol, suggesting that the ligand achieved a more energetically favorable binding conformation throughout the simulation. The calculated MM/GBSA binding free energy of compound 7b improved from −38.39 to −45.04 ± 4.28 kcal/mol after MD simulation, indicating a more favorable predicted protein‐ligand interaction. The MM/GBSA binding free energy of compound 7d improved from −41.36 to −49.92 ± 6.31 kcal/mol. The reference ligand, remdesivir, demonstrated a good improvement, with the binding free energy decreasing from −22.33 to −64.95 ± 4.92 kcal/mol, highlighting considerable stabilization during the MD. The energy decomposition analysis indicated that the binding of compound 7a was primarily driven by van der Waals interactions (−45.1587 kcal/mol), lipophilic forces (−10.6799 kcal/mol), and contributions from hydrogen bonding (−4.7013 kcal/mol). The individual energetic components contributing to the binding free energies of the respective compounds are summarized in Table 4. The findings from the post‐MD MMGBSA assessment indicate that compound 7a exhibited a more favorable predicted MM/GBSA energetic profile after MD simulation compared with the pre‐MD binding free energy, energetic profile, supporting its computational prioritization for further experimental evaluation.

**TABLE 4:** The binding free energy along with contributing energies of the lead candidates.

Drug leads	Binding free energy (kcal/mol)	van der Waals energy (kcal/mol)	Coulomb energy (kcal/mol)	Covalent (kcal/mol)	Lipophilic energy (kcal/mol)	H bond (kcal/mol)
Remdesivir	−64.9529 ± 4.92	−62.3010	−38.7086	1.4528	−12.9872	−4.8230
Compound 7b	−45.0405 ± 4.28	−38.9905	22.1878	1.8452	−8.7100	−3.9114
Compound 7d	−49.9271 ± 6.31	−53.2395	15.1630	2.9177	−13.2930	−4.4090
Compound 7a	−53.7347 ± 4.08	−45.1587	12.0251	3.4128	−10.6799	−4.7013

The residue‐wise MM/GBSA analysis indicated favorable energetic contributions from multiple residues involved in the ligand‐protein interactions across 7a, 7b, 7d, and remdesivir (Figure 12). The contributing residues comprised those within or adjacent to the conserved QGDNQ region, together with other interacting residues in the binding pocket. Notable favorable contributions were observed for Lys598, Arg529, Trp599, and Asn705. Trp599 showed a favorable contribution in all the complexes, whereas Asn705 and Asp704 contributed energy values of −2.87 and −3.53 kcal/mol for 7d and remdesivir, respectively, with Asn705 contributing −5.17 kcal/mol in the remdesivir complex. Overall, the decomposition profiles of the residues offered further energetic contribution of the predicted active‐site region and the interacting residues in the ligand‐bound complexes.

**FIGURE 12:**
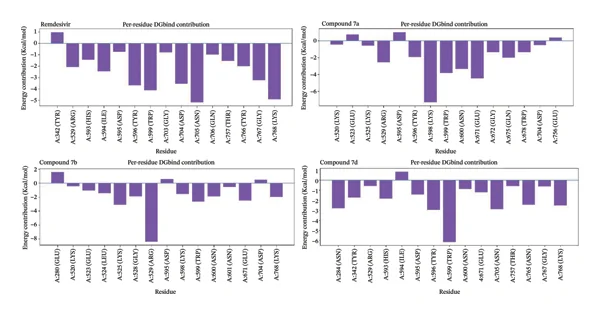
Residue‐wise MM/GBSA decomposition profiles of the remdesivir and compound 7a, compound 7b, and compound 7d.

#### 3.5.5. PCA

PCA is a statistical technique used to determine and rank the main collective movements of a protein system, ranging from the most significant to the least significant. This method successfully reduces the dimensions required to describe protein dynamics [54]. The protein‐only (apo) RdRp showed a broader conformational distribution, indicating its inherent flexibility and increased conformational sampling in the absence of ligand binding. The contributions of PC1 and PC2 were 22.493% and 14.502% for the protein‐only system, 22.799% and 17.285% for compound 7a, 27.992% and 12.032% for compound 7b, 35.804% and 8.254% for compound 7d, and 26.841% and 8.756% for remdesivir. Quantitative analysis revealed that the first two principal components accounted for 36.995% of the total variance in the protein‐only system, compared with 40.083%, 40.025%, 44.059%, and 35.598% for compounds 7a, 7b, and 7d and remdesivir, respectively. The variance contributions of the principal components for all systems are provided in Supporting Table 1-D. The PC1‐PC2 projections therefore provide a quantitative basis for comparing the major conformational motions sampled during the 200 ns simulations. As shown in Figure 13, remdesivir formed a relatively compact conformational cluster with moderate dispersion, indicating relatively compact conformational behavior throughout the simulation. Among the screened compounds, compound 7a exhibited a relatively compact and well‐defined conformational distribution similar to that of the reference complex and more restricted than the protein‐only system. This pattern indicates differences in the conformational distributions of the ligand‐bound systems relative to the protein‐only system. All complexes remained largely confined to well‐defined conformational regions throughout the 200 ns simulation, without evidence of substantial conformational drift. The results showed that compound 7a exhibited a relatively compact conformational distribution compared with the other screened compounds.

**FIGURE 13:**
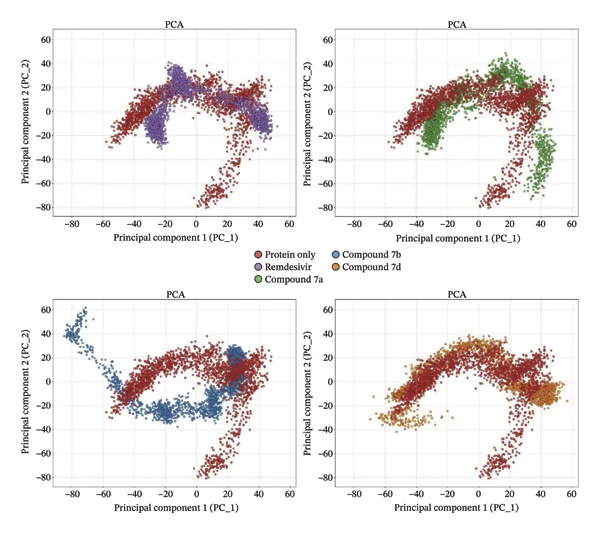
Principal component analysis (PCA) of apo CHPV RdRp and its complexes with compounds 7a, 7b, and 7d and remdesivir.

## 4. Discussion

CHPV, a member of the Rhabdoviridae family, possesses a negative‐sense, single‐stranded RNA genome [55]. It is a recently discovered pathogen associated with severe encephalitis in children, with reported case fatality rates between 56% and 75% [2]. The occurrence of periodic outbreaks and the lack of approved therapeutics highlight the need to explore novel antiviral targets. The current study employed a comprehensive computational approach to discover potential inhibitors that target the CHPV’s RNA‐dependent RNA polymerase, with an emphasis on the conserved catalytic QGDNQ motif. Homology modeling was utilized to create a structural model of the protein. The generated model demonstrated good reliability supported by high sequence identity with the selected template, favorable quality assessment scores, and stable behavior during the MD simulation. The presence of the conserved QGDNQ motif within the predicted active site highlights its functional importance in RNA synthesis, consistent with its conservation among rhabdoviral polymerases. Computational analysis of polyphenolic natural compounds against the SARS‐CoV‐2 spike protein, as well as crucial nonstructural proteins, identified substances with possible interactions at targets related to viral entry and replication. This highlights the usefulness of in silico screening for prioritizing natural products for further experimental evaluation [56]. Coumarins are naturally occurring benzopyrone derivatives with diverse chemical structures and broad pharmacological activities, making them attractive scaffolds for medicinal chemistry and antiviral drug discovery. The ability of coumarin derivatives to interact with different viral enzymes has been demonstrated against HIV, hepatitis viruses, influenza virus, dengue virus, and chikungunya virus, and the current results further broaden their potential use in inhibiting rhabdoviral RNA polymerases [21–24]. Similarly, computational repurposing of natural products for targeting viral attachment and entry‐associated targets has demonstrated the broader application of structure‐based screening for prioritizing potential antiviral candidates [57].

Remdesivir was selected as the reference ligand based on its reported antiviral activity against CHPV and its previous use as a reference compound in computational studies of CHPV, including molecular docking and MD simulations [9, 43]. This approach is also consistent with previous molecular docking studies of related Mononegavirales RdRp systems, where remdesivir was used as a reference/control compound [41]. The active triphosphate metabolite acts through incorporation into nascent viral RNA and subsequent inhibition of polymerase progression, a mechanism that is not fully captured by conventional molecular docking. Therefore, the remdesivir docking results in the present study should be interpreted as a comparative computational benchmark rather than a direct representation of its pharmacological mechanism of action.

The virtual screening of the synthesized coumarin‐triazole derivatives identified compounds 7a, 7b, and 7d as the most promising candidates based on their docking scores and calculated MM/GBSA binding free energies. All three compounds demonstrated promising interactions within the catalytic pocket; however, compound 7a displayed the most favorable docking affinity and established several interactions with residues in the vicinity of the catalytic motif, especially Asn705 and adjacent active‐site residues. The relatively favorable predicted docking score of compound 7a compared with the reference ligand supports further investigation of the coumarin‐triazole framework as a potential scaffold for CHPV RdRp inhibitor development.

Initial pharmacokinetic assessment provided further computational information on the selected compounds. The ADMET analysis showed variations across the evaluated physicochemical, absorption, distribution, metabolic, and toxicity‐related endpoints, with several parameters falling within the reference ranges. These findings support the use of ADMET prediction as a preliminary computational assessment during compound prioritization. Hydroxylated coumarin derivatives can experience metabolic changes, including oxidation and conjugation, which may influence their metabolic stability and overall systemic exposure. The incorporation of a 1,2,3‐triazole moiety in the present compounds may provide chemical stability advantages because of the reported stability of the triazole ring toward hydrolytic and oxidative/reductive conditions [58, 59]. Nonetheless, the current computational analysis does not directly confirm metabolic stability or show that the triazole modification inhibits metabolic degradation. Experimental metabolic stability studies are therefore required to determine whether this modification provides an advantage over the corresponding parent coumarin structures. These computational findings may serve as supportive evidence for advancing the prioritized compounds to subsequent in vitro safety and efficacy studies.

Even with these computational findings, the translational potential of the selected compounds needs additional exploration. As highlighted by Dsouza (2026), promising computational and preclinical findings may fail to translate into clinical development because of pharmacokinetic, metabolic, safety, and other biological limitations. In the present study, the predicted hepatic clearance values (40.80–50.61 μL·min^−1^ (10^6^ cells)^−1^) indicate measurable hepatic clearance, however, these predictions alone cannot determine the degree of hepatic first‐pass metabolism. Similarly, the predicted BBB penetration values (10.86%–16.70%) provide preliminary information on potential CNS distribution yet do not indicate therapeutically relevant brain exposure, an essential factor given the neurological symptoms linked to CHPV infection. Furthermore, the expected values for oral bioavailability (ranging from 43.87% to 46.11%) should not be seen as proof of therapeutic effectiveness, since there is no universally accepted standard that determines what constitutes adequate oral bioavailability. As a result, experimental investigations focusing on metabolic stability, pharmacokinetics, CNS penetration, cytotoxicity, and antiviral efficacy are essential to evaluate if the computationally prioritized compounds can address these possible translational challenges [60].

MD simulations were subsequently performed to evaluate the stability of the protein‐ligand complexes under dynamic physiological conditions. The protein‐only system exhibited a stable structural configuration throughout the 200 ns simulation and served as a reference for assessing ligand‐associated changes in protein dynamics. RMSD and RMSF analyses showed broadly comparable profiles among the ligand‐bound systems, with no substantial structural deviations. Among the compounds, compound 7a consistently demonstrated greater structural stability, exhibiting lower RMSD and RMSF values relative to compounds 7b and 7d, suggesting relatively lower conformational fluctuations in the 7a‐bound system. Protein‐ligand interaction analysis provided further support for the favorable binding behavior of compound 7a. Throughout the simulations, compound 7a maintained hydrogen‐bonding and hydrophobic interactions with residues surrounding the conserved QGDNQ motif, including Asn705 and neighboring residues within the predicted polymerase binding region.

The subsequent MM/GBSA calculations showed that MD refinement resulted in more favorable predicted binding energetics for the ligand. The improved MM/GBSA binding free energy observed for compound 7a after the MD simulation suggests that structural relaxation enabled the complex to adopt a more energetically favorable conformation. Although Remdesivir showed a more favorable binding free energy after the MD simulation, this observation should be interpreted cautiously, as remdesivir is a nucleotide prodrug that requires metabolic activation to its triphosphate form inside the cell before it can inhibit viral polymerases. Consequently, comparisons with remdesivir should be regarded as computational benchmarks rather than direct assessments of mechanisms since the two classes of compounds inhibit RdRp via different processes.

The PCA analyses provided additional qualitative information on the collective dynamic behavior of the systems. PCA indicated variations in the conformational distributions of the complexes, with compound 7a displaying a relatively compact conformational distribution. Among the assessed derivatives, compound 7a demonstrated relatively favorable predicted docking and MM/GBSA results, together with the observed interaction profile with residues surrounding the conserved QGDNQ motif. Overall, the computational analyses supported the prioritization of compounds 7a, 7b, and 7d for further investigation, with compound 7a showing several favorable computational characteristics. However, these findings represent computational predictions and require experimental validation to determine their antiviral activity and inhibitory effects against CHPV RdRp.

Future research should assess the prioritized coumarin‐triazole compounds through biochemical assays of CHPV RdRp inhibition using recombinant polymerase to ascertain IC_50_ values [61]. This could be followed by cytotoxicity assessment [62], as well as assays for CHPV‐infected cells or replicons to ascertain CC_50_ and EC_50_ values alongside selectivity indices. Appropriate concentration‐response curves and assay controls should be used for reliable EC_50_ estimation [63]. Analyzing the mutations of residues in or near the conserved QGDNQ motif that engages with the prioritized compounds may provide additional insight into their role in compound recognition and the inhibition of RdRp. These findings establish a solid basis for the systematic development of non‐nucleoside inhibitors targeting CHPV and support additional optimisation of the coumarin‐triazole framework for the discovery of antiviral drug discovery.

## 5. Conclusion

In this study, a comprehensive computational strategy was employed to discover potential inhibitors of the RdRp of the CHPV by targeting its conserved catalytic QGDNQ motif. Among the screened coumarin‐triazole derivatives, compound 7a consistently demonstrated the relatively favorable predicted docking score, stable interactions between the protein and ligand, enhanced binding free energy after MD simulation. The RMSD, RMSF, and PCA analyses provided qualitative insights into the dynamic behavior of the ligand‐bound systems during the 200 ns simulation. The results indicate that compound 7a is a promising lead candidate for the development of non‐nucleoside inhibitors against CHPV RdRp. Nonetheless, these results rely exclusively on computational predictions and require experimental validation through biochemical RdRp inhibition assays, cytotoxicity assessment, and cell‐based antiviral research to determine their actual antiviral activity and therapeutic potential.

## Author Contributions

Anjana C. Lalu: writing–review and editing, writing–original draft, methodology, investigation, formal analysis, conceptualization, and visualization. Varun Thachan Kundil: writing–review and editing, formal analysis, and investigation. Mukhtar Ahmed: writing–review and editing and formal analysis. Bristow Ben Joseph: review and editing and visualization. Amritha Thaikkad: writing–review and editing and formal analysis. Radul R. Dev: writing–review and editing and formal analysis. Muthipeedika Nibin Joy: writing–review and editing and formal analysis. Rajesh Raju: writing and review and editing, conceptualization, and supervision. Abhithaj Jayanandan: writing–review and editing, conceptualization, and supervision. Grigory V. Zyryanov: writing–review and editing and formal analysis.

## Funding

Dr. Mukhtar Ahmed is supported by the Ongoing Research Funding Program (ORF‐2026‐984), King Saud University, Riyadh, Saudi Arabia. Grigory V. Zyryanov is thankful to the Russian Science Foundation (Grant # 25‐73‐30016) for financial support.

## Ethics Statement

The authors have nothing to report.

## Consent

It is hereby confirmed that all authors are aware of the contents of this manuscript and provide consent for its publication.

## Conflicts of Interest

The authors declare no conflicts of interest.

## Supporting Information

Additional supporting information can be found online in the Supporting Information section.

## Supporting information


**Supporting Information** (1) Supporting Tables 1‐A and 1‐B: summarize the binding‐site residues predicted by SiteMap and P2Rank, respectively. (2) Supporting Table 1‐C lists the docking scores and SMILES representations of the 80 screened coumarin‐triazole derivatives. (3) Supporting Table 1‐D: the principal‐component variance contributions for the apo protein and ligand‐bound systems. (4) Supporting File 1 provides the proposed synthetic procedures and reaction conditions for the preparation of intermediates 3 and 4, intermediates 6a–d, and the final coumarin‐triazole hybrids 7a–d.

## Data Availability

The data that support the findings of this study are available from the corresponding author upon reasonable request.
