# Supplementary material for: Structure‐Based Discovery of Coumarin‐Triazole Derivatives Targeting the Conserved QGDNQ Motif of Chandipura RdRp
Source: Scientifica (Cairo). 2026 Nov 10;2026:1713642. doi: 10.1155/sci5/1713642 (PMC13749632; doi:10.1155/sci5/1713642)
Supplement: Supplementary file 1 [file SCI5-2026-1713642-s001.zip › Supplementary file 1 .docx]

**Supplementary file 1:The synthetic procedures and reaction conditions**

**Experimental procedure for the synthesis of intermediate 3**

The synthesis of intermediate 3 will be carried out by a previously reported procedure <https://doi.org/10.1063/5.0018033> . Briefly, resorcinol (1 equiv.) will be reacted with concentrated H₂SO₄ in a two-necked round-bottomed (RB) flask and will be cooled to 0 °C. To this mixture, diethyl-1,3-acetonedicarboxylate (1.2 equiv.) will be added dropwise, and the reaction mixture will be gradually warmed to room temperature and will be stirred for 15 hours. The progress of the reaction will be monitored by TLC. After completion of the reaction, the mixture will be poured into ice-cold water, and the resulting precipitate will be filtered, washed with water, and dried to obtain the crude product. The resulting crude product will be recrystallized from ethanol to obtain intermediate compound 3.

**Experimental procedure for the synthesis of intermediate 4**

The synthesis of intermediate 4 can be carried out by a previously reported procedure <https://doi.org/10.24820/ark.5550190.p012.480> . To the weighed quantity of coumarin intermediate 3 (1 equiv.) in acetone, K₂CO₃ (3 equiv.) and propargyl bromide (1.2 equiv.) will be added under an inert atmosphere, and the reaction mixture will be stirred at room temperature for 12 hours. The completion of the reaction will be monitored by TLC, and the reaction mixture will be poured into ice-cold water with vigorous stirring. The solution will be extracted with ethyl acetate, and the organic layer will be separated, washed with brine, dried over Na₂SO₄, and concentrated under reduced pressure to obtain intermediate compound 4.

**Experimental procedure for the synthesis of intermediates 6a-d**

The synthesis of intermediates 6a-d can be carried out by a previously reported procedure <https://doi.org/10.1070/RCR5042> . To a solution of intermediate 4 (1 equiv.) in 1,2-dichloromethane, azides 5a-d (1 equiv.), CuI (0.1 equiv.), and DIPEA (1.5 equiv.) will be added, and the reaction mixture will be stirred at room temperature for 8 hours. After completion of the reaction, as monitored by TLC, the reaction mixture will be quenched with water and will be extracted with 1,2-dichloromethane. The organic layer will be dried over Na₂SO₄ and will be concentrated under reduced pressure to obtain the crude product. The crude product will be further recrystallized from ethanol to obtain intermediates 6a-d.

**Experimental procedure for the synthesis of final compounds 7a-d**

The synthesis of final compounds 7a-d will be carried out by a previously reported procedure <https://doi.org/10.1063/5.0018033> . To a weighed quantity of intermediates 6a-d (1 equiv.) in a two-necked round-bottomed (RB) flask containing ethanol, NaOH (1.2 equiv.) will be added, and the reaction mixture will be stirred at room temperature for 2 hours. After completion of the reaction, as monitored by TLC, the reaction mixture will be poured into ice-cold water and will be acidified with concentrated HCl. The resulting precipitate will be filtered, washed with cold water, and dried. The residue will be further recrystallized from ethanol to obtain the entitled final compounds 7a-d
